# Analysis of Different Organic Rankine and Kalina Cycles for Waste Heat Recovery in the Iron and Steel Industry

**DOI:** 10.1021/acsomega.2c03922

**Published:** 2022-12-06

**Authors:** Davood Atashbozorg, Afshin Mohseni Arasteh, Gholamreza Salehi, Masoud Torabi Azad

**Affiliations:** † Energy System Engineering Department, North Tehran Branch, Islamic Azad University, Tehran165, Iran; ‡ Department of Physical Oceanography, North Tehran Branch, Islamic Azad University, Tehran165, Iran; § Mechanical Engineering Department, Central Tehran Branch, Islamic Azad University, Tehran165, Iran

## Abstract

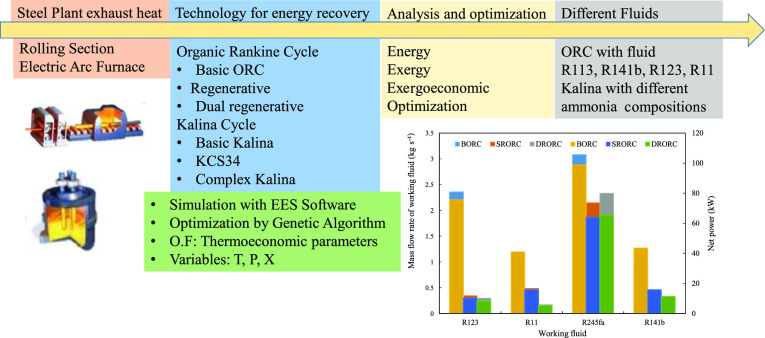

This study analyzed waste heat of two sections including the rolling section and electric arc furnace with low and medium temperature ranges, respectively. Organic Rankine cycles (ORCs) and Kalina cycles are the best technologies for the conversion of low-quality and medium-quality thermal energy to electrical power. The ORC applies the principle of the steam Rankine cycle, but it uses organic working fluids with low boiling points to recover heat from lower temperature heat sources. Also, in the Kalina cycle, ammonia water is selected as the working fluid because of its variable boiling point and thermodynamic properties. This study employs the thermo-economic method using the genetic algorithm to optimize the performance of three different ORC systems including a basic ORC (BORC) system, a single-stage regenerative ORC (SRORC) system, and a double-stage regenerative ORC (DRORC) system using five different working fluids and a basic Kalina cycle with KCS34 and complex cycle under the same waste heat conditions. Based on the energy and exergy analysis, the complex Kalina cycle shows the best performance among all studied cycles. The next best performance was exhibited by KCS34 and DROC, respectively. In general, Kalina cycles and ORCs are suitable for low-temperature and medium-temperature heat sources, respectively. According to the thermo-economic analysis, KCS34 in the rolling section and DRORC in EAF show optimum performance for heat recovery. R11 and R113 are selected as the best working fluids for ORCs, and ammonia with a concentration of 0.9 in the mixture is the optimal solution for Kalina cycles.

## 1 Introduction

The steel industry has the second-largest heat recovery potential followed by the oil and gas industry.^1^ In the steel industry, a large amount of excess heat is generated since its production process is often conducted at high temperatures. Huge amounts of heat are wasted in some sections of the steel industry. The organic Rankine and Kalina cycles are the most common technologies for waste recovery in this temperature range. With these technologies, site efficiency can be increased and power can be produced for use in different parts of industry that need electricity. Nguyen et al.^2^ investigated power generation from residual industrial heat. In this research, they investigated methods of recovering the residual low-grade thermal energy and converting it into higher quality mechanical energy using the principle of the thermodynamic Rankine cycle. Xi et al.^3^ analyzed and optimized the regenerative organic Rankine cycle (ORC) for waste heat recovery using a genetic algorithm. By using exergy efficiency as an objective function, the performance of three different ORC systems, including a basic ORC (BORC) system, a single-stage regenerative ORC (SRORC) system, and a double-stage regenerative ORC (DRORC) system, which used six different working fluids under the same waste heat condition, was examined.

Mohammadkhani et al.^4^ conducted an exergoeconomic assessment and a parametric study on a gas turbine-modular helium reactor combined with two ORCs. A parametric study was also carried out to reveal the effects on the exergoeconomic performance of the combined system of such significant parameters as compressor pressure ratio, turbine inlet temperature, temperatures of evaporators, pinch point temperature difference in the evaporators, and degree of superheat at the inlet of the ORC turbines. In the end, the combined cycle performance was optimized exergoeconomically. The results showed that the precooler, intercooler, and ORC condensers exhibited the worst exergoeconomic performance. Imran et al.^5^ reviewed the literature on the thermo-economic optimization of BORC and regenerative ORC for waste heat recovery applications under constant heat source conditions. The thermal efficiency and specific investment cost of BORC, SRORC, and DRORC were optimized by using a Non-dominated Sorting Genetic Algorithm-II (NSGA-II). Anvari et al.^6^ demonstrated thermo-economical considerations of regenerative ORC coupled with absorption chiller systems incorporated in a trigeneration system. This system consists of three sections of gas turbine and heat recovery steam generator cycle, regenerative ORC, and absorption refrigeration cycle. Arslan^7^ investigated the Kalina cycle used for electricity generation from medium-temperature geothermal resources. The optimum operating conditions for the KCS34 plant design were determined based on the exergy and economic concepts.

Zhang et al.^8^ reviewed research on Kalina cycles including the description of the Kalina cycle, the comparison of the Rankine and Kalina cycles, energy and exergy analysis on the Kalina cycle, different Kalina systems, and their different applications. Their paper is concluded with a discussion on some techniques concerning the ammonia–water mixture, including stability, environmental impacts, safety, and corrosion problems.

Chen et al.^9^ performed an energy and exergy analysis on an integrated system of the ammonia–water Kalina–Rankine cycle, which is a novel cycle operated on the Kalina cycle for power generation in nonheating seasons and on the ammonia–water Rankine cycle for cogeneration of power and heating water in winter. Bahrampoury and Behbahaninia^10^ investigated the thermodynamic optimization and thermo-economic analysis of four double-pressure Kalina cycles driven by Kalina cycle system 11. In this study, the heat transfer fluid of the inlet stream is supposed to be the product of combustion at three different temperatures, 383.15, 413.15, and 443.15 K. The results are compared at the base case and under optimal conditions. Kaşka^11^ performed an energy and exergy analysis on a waste heat-driven ORC using actual plant data of a steel factory. There are many furnaces in steel factories, and they are used to make steel slabs soft enough to roll. One of the heat rejection processes in the furnaces is the cooling of walking beams that are used to carry slabs through the furnaces. Water is used to cool these walking beams, and the temperature of the cooling water leaving the furnace in this case study is 122.4 °C. Two indexes contain thermosustainability, and optimum exergetic performance for ORCs was developed by Abam et al.^12^

Sung et al.^13^ analyzed the performance of a 200 kW ORC system that is used in the steel processing plant. The real-time operating characteristics of the ORC system are demonstrated with actual flue gases, and an ORC system with R245fa refrigerant was developed for a heat source temperature of 140 °C. The evaporation and condensation pressures were 2.090 and 220 kPa, respectively, and the net power output was 235.7 kW with a thermal efficiency of 12.9%. Zhang et al.^14^ investigated waste energy recovery and energy efficiency improvement in China’s iron and steel industry. The iron and steel industry is one of the most energy-intensive manufacturing industries and consumes large amounts of primary energy as waste energy. Ren et al.^15^ studied greenhouse gases and carbon emission reduction technologies in the iron and steel industry using life cycle analysis. They found that CO_2_ emission can be reduced by 43% using advanced technologies, and this can be increased to as high as 80% if super-advanced technologies can be achieved. Yun et al.^16^ explored CO_2_ capture in the iron and steel technology with a focus on absorption and membrane technologies. They reported that the absorption technology had lower efficiency than the membrane technology.

Mass and energy balance was explored by Sun et al.^17^ in the steel manufacturing industry. They examined numerous steel production methods and literature and addressed the technologies for the reduction of energy use. Bailera et al.^18^ analyzed power to X processes in the steel industry in which X can represent iron, hydrogen, syngas, methane, and methanol. Their research introduced the integration of oxy-fuel iron-making and power to gas processes. Su et al.^19^ studied energy recovery in 12 industries in three categories. The research dealt with different heat sources of different technologies and their thermal performance, profitability, and environmental impacts. He and Wang^20^ reviewed the technologies that were appropriate for energy consumption, the improvement of efficiency, and the reduction of energy use. The steel industry is an energy-intensive industry so that it accounted for 18% of the energy consumption of the industrial sector in 2013. This energy consumption can be reduced by 20% using advanced technologies.

Wang et al.^21^ conducted a water-energy-emission nexus analysis in China’s steel industry. The sintering section had the highest energy consumption rate of about 57%, and the rolling section had the highest water consumption rate of 31%. They also estimated the pollutants emitted from different sections. A simultaneous analysis of water and energy was performed by Gao et al.^22^ in three technology categories to reduce energy consumption and direct and indirect water consumption. Egilegor et al.^23^ studied three different industries, including aluminum, tile, and steel, and considered the possibility of using thermal tubes. The steel industry studied in this paper had waste temperatures of about 200–450 °C and a mass flow rate of 1000–8000 kg/h wasted hot gas. The recovery potential of this industry could be considered to be 620 kW, 40% of which can be realized by using the thermal tube technology. Lecompte et al.^24^ conducted a case study on the use of ORC for an electric arc furnace. In their analysis, it would be possible to generate 752 kWe with 25 bar steam for a 100 MWe furnace. In the case of simultaneous generation, a power of 521 kWe with 4.52 MW heat would be possible. Khosravi et al.^25^ addressed different arrangements of the Rankine cycle for heat recovery from a gas turbine. Wang et al.^26^ studied different technologies affecting energy consumption from the perspective of the mitigation of initial energy consumption or waste heat recovery and its conversion to consumed heat or electricity or cooling generation.

One method to prevent heat recovery system performance and power generation under the variable temperature of the heat waste source and its variable load is to employ a compressed water heat storage system, which was studied by Couvreur et al.^27^ ORC was first used in the steel industry in Singapore. Then, Campana et al. investigated the feasibility of its application in the steel industries of the European Union. They made analyses in two separate sections of EAF and rolling. Biondi et al.^28^ studied the feasibility of using the CO_2_ cycle for heat recovery in the steel manufacturing process. The overall efficiency was estimated at 30% and the capital return at 4.5 years. The feasibility study of using energy wasted by ORC in Germany’s steel industry by Pili et al.^29^ showed that the BOF section had the highest possibility of power generation followed by the EAF and RHF sections, respectively. Ortega-Fernández and Rodríguez-Aseguinolaza^30^ investigated the feasibility of waste heat storage in the steel industry. Kaşka^11^ conducted an energy and exergy analysis on ORC for power generation in the steel industry.

**Figure 1:**
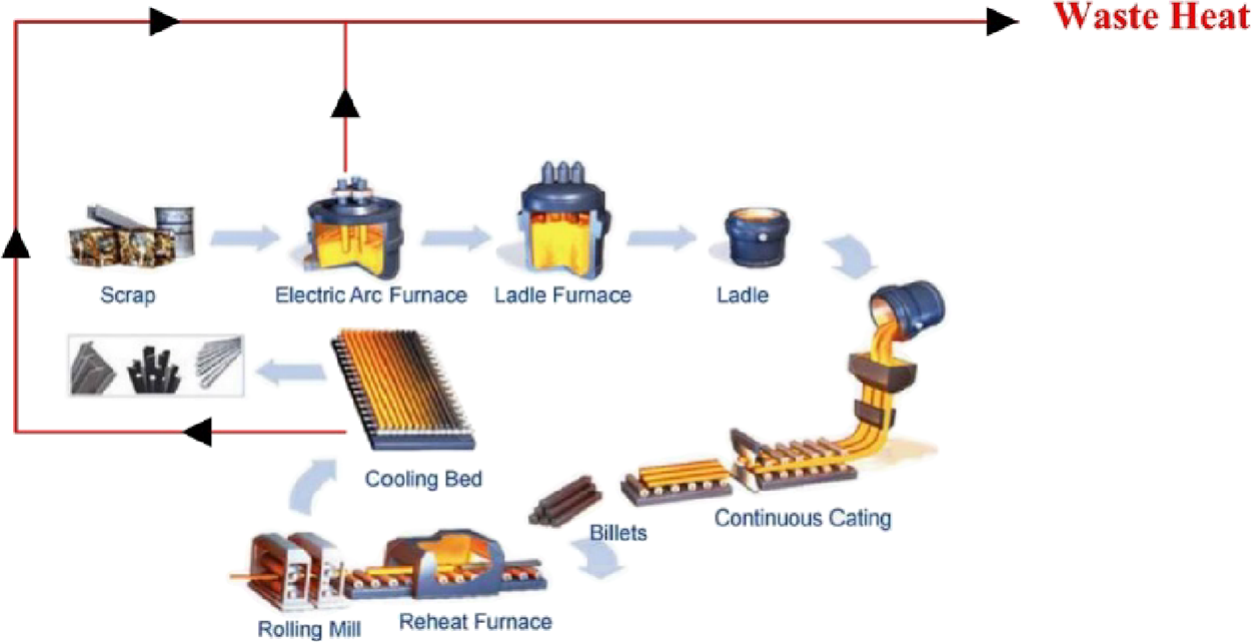
Waste heat from a steel plant.

In the steel industry, a large amount of excess heat is generated and wasted to the environment, which affects GWP and ODP (Figure 1). By recovering waste heat, the efficiency of the system can be improved, which will result in the reduction of fuel consumption by the industry on the one hand, and greenhouse emissions will decrease, resulting in less air pollution and a lower increase in temperature on the other hand. In many parts of the iron and steel industry, one can see sensible heats that have the potential to be recovered, for example, in coke oven, sintering machine, blast furnace, BOF, EAF, rolling process, and so on. ORC and Kalina cycle technologies have been designed for these reasons. By these cycles, we can recover the waste heat of the industry and produce power to use on-site. Previous investigations on simulating and analyzing ORCs and Kalina cycles have not considered this sector. We selected three different models of ORCs and three different models of Kalina cycles for the present investigation. The research aims to analyze energy and exergy, as well as computerized optimization, to find optimal operating parameters. Also, optimization by the genetic algorithm is used to test different working fluids to find ideal conditions for maximum efficiency and power output within certain constraints. Then, an exergoeconomic analysis is performed on the system to determine electricity generation costs using different working fluids. Finally, Kalina cycles and ORCs are compared to find out the best application to recover waste heat for a specific temperature range that has been identified in the steel production process.

The specific objectives of this research are listed below:

- Analyzing the steel production process to find heat-wasting sections.
- Using waste heat as an appropriate heat source for the evaporator of the organic Rankine and Kalina cycles to recover for power production in the system, improve the efficiency, and reduce environmental pollution.
- Selecting different working fluids in ORC to find out fluids with better efficiency in different conditions.
- Analyzing energy and calculating the overall efficiency for each cycle to evaluate performance and alleviate environmental pollution.
- Analyzing exergy and optimization of exergy efficiency to find the components with higher exergy destruction in ORC and Kalina cycles.
- Performing a parametric study of the system using various parameters to observe efficiency behavior (energy and exergy) and use the study for comparison against optimization results.
- Optimization and thermo-economic investigation for the systems to evaluate the cost rate of electricity.
- Comparing different systems and selecting the best cycle for waste heat recovery for a specific temperature range identified in the steel production process.

Our goal in this research is to recover waste heat for power production in the steel industry. We carefully observed the site of steel production and recognized sections with a significant potential for heat recovery. Then, selections were made from two different waste heat sites in the steel production process, i.e., the rolling and electric arc furnace sections. One is in the medium-temperature range, and the other is in the low-temperature range. In this research, we analyze three different ORCs and three different Kalina cycles to find out the best system for heat recovery on the target temperature range.

## 2 Description of Research Cycles and Energy and Exergy Analysis

### 2.1 ORC and Kalina Cycles of Study

As we mentioned, the following three ORCs were selected for this study:

- BORC (basic organic Rankine cycle)
- SRORC (single regenerative organic Rankine cycle)
- DRORC (double regenerative organic Rankine cycle)

**Figure 2:**
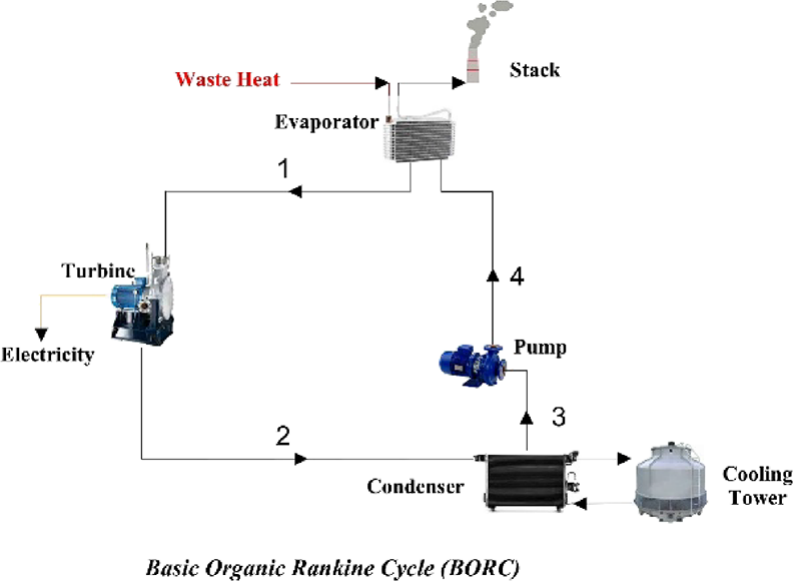
Basic organic Rankine cycle (BORC).

**Figure 3:**
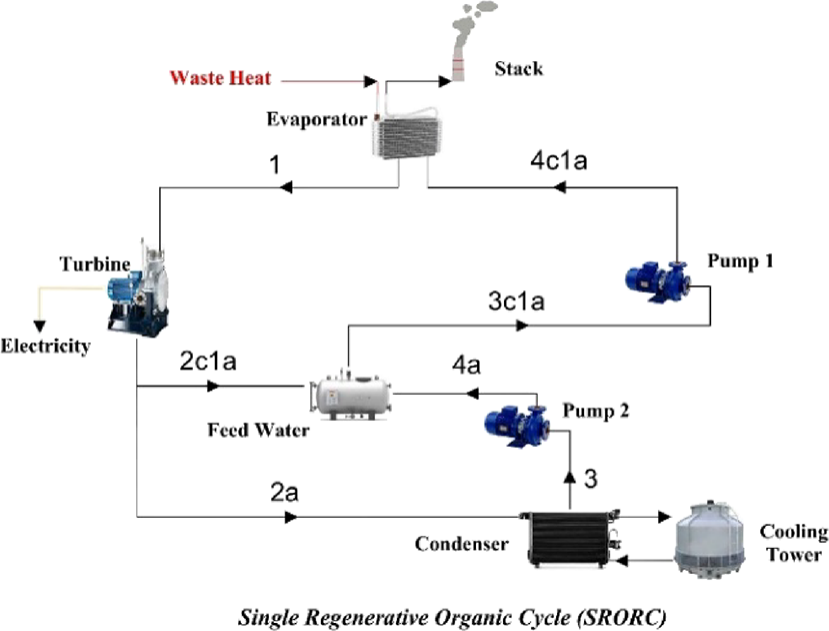
Single regenerative organic Rankine cycle (SRORC).

**Figure 4:**
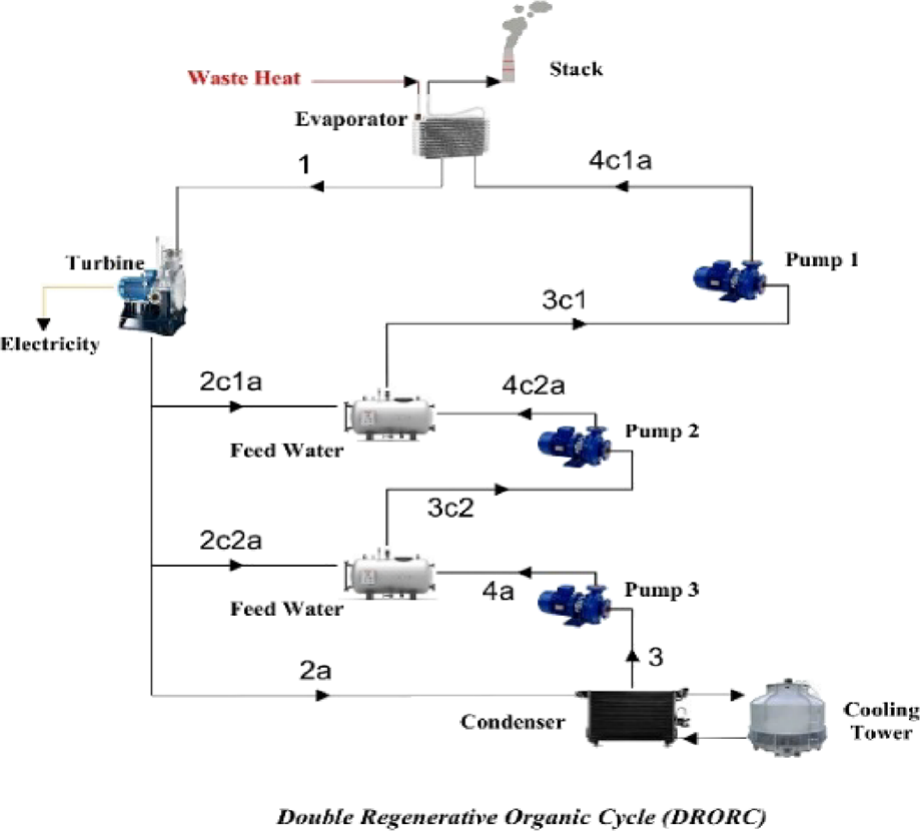
Double regenerative organic Rankine cycle (DRORC).

The schematics of these cycles are depicted in Figures 2–4.

**Figure 5:**
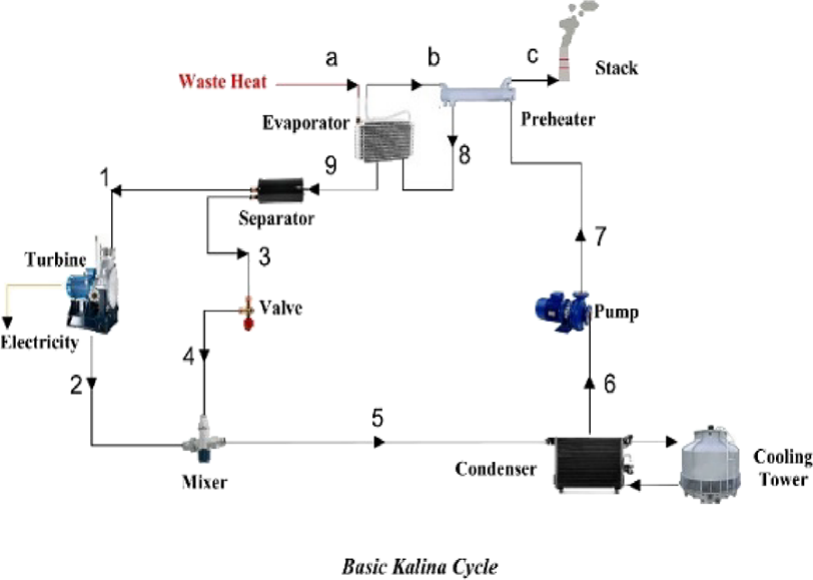
Basic Kalina cycle.

**Figure 6:**
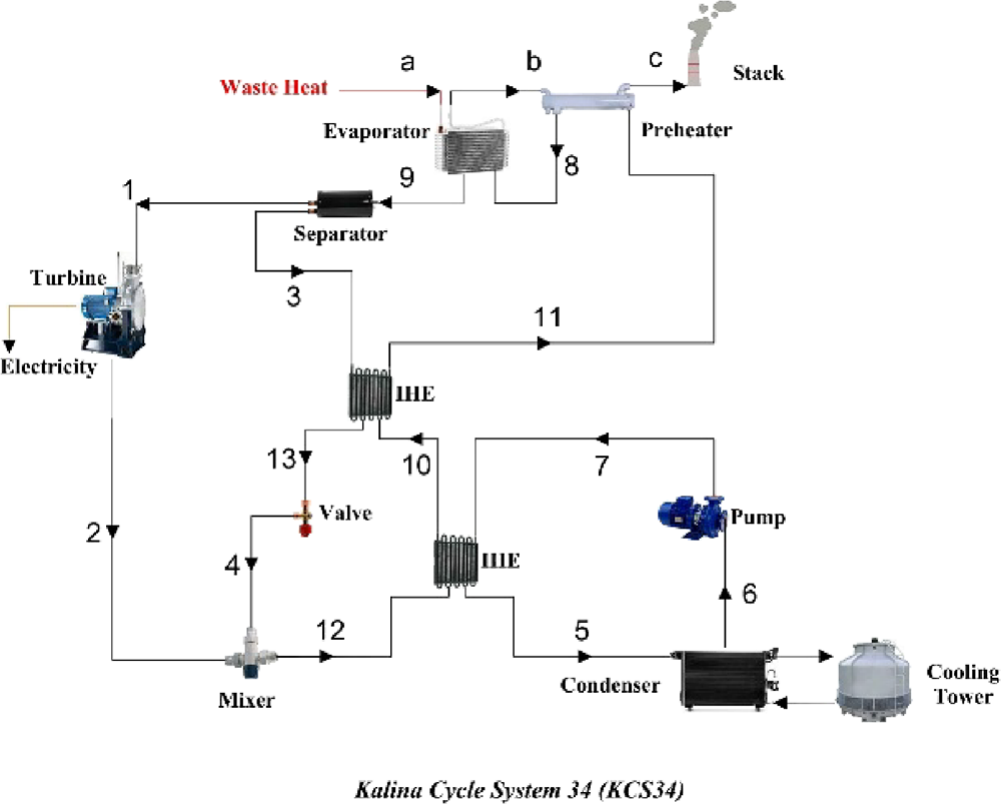
Kalina cycle system 34 (KCS34).

**Figure 7:**
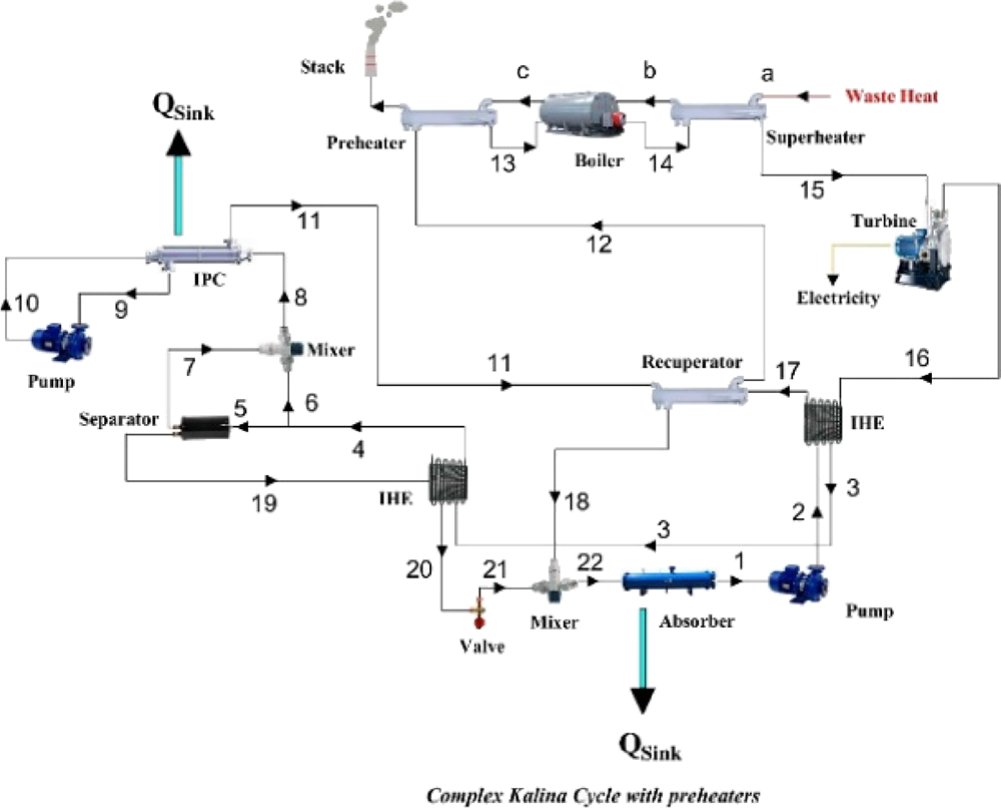
Complex Kalina cycle with preheaters.

BORC is a basic form of the ORC. To improve the performance of ORCs, different modified cycles, such as regenerative ORCs, have to be analyzed and compared with the basic ORC. As seen in Figures 3 and 4, regenerative ORCs differ from BORC in the sense that vapor is divided into several parts (two parts in SRORC and three parts in DRORC). Then, parts of the vapor go into the feed-water heaters where they act as preheaters before the evaporator to improve the performance of the ORC system. By this method, efficiency is expected to increase versus the basic type. To deepen the research and get better results, we chose three cycles in the Kalina type, which include the basic Kalina cycle, Kalina cycle system 34 (KCS34), and complex Kalina cycle with preheaters. You can see the schematic of these three cycles in Figures 5–7. As is evident in Figure 6, the difference between the BORC and the Kalina cycle is that Kalina has a separator and a mixer to recover more heat by changing the concentration of ammonia–water in the boiler. KCS34 is suitable for temperatures below 250 °F (121 °C). The separator in the cycle ensures that the vapor is only directed to the turbine. The KCS34 design has a recuperator in the turbine exhaust stream prior to the condenser. The complex Kalina cycle uses an additional mixing process that results in four different ammonia concentrations in the cycles where XV < XB < XWF < XV in terms of the ammonia concentration and XWF is the working fluid concentration.

### 2.2 Steel Industry Data

As discussed in Section 2, our goal is to recover medium- and low-grade waste heat in the steel industry by the introduced cycles. So, we selected three different sections of the steel production process with two different temperatures and flow rate ranges to find the best cycle for each process. Table 1 presents the selected sensible heat with their conditions.

**Table 1:** Temperature and Flow Rate of Waste Heat

sensible heat	temperature (K)	flow rate (kg s^–1^)	specific heat at constant pressure (kJ kg^–1^ K^–1^)
rolling section	420	14	1.1
electric arc furnace	520	38	1.1

### 2.3 Simulation Assumptions

Energy analysis aims to determine the thermodynamic efficiency of the cycles and to compare them to find out the best system for recovery. Thermodynamics has two important and basic laws: the first and second laws. The first law analyzes the conservation of energy in the process, while the second law is used to discuss the quality of energy and material.

To simulate cycle performance in this study, the following assumptions are employed:

- All processes in the cycles are assumed to be at the steady state and steady flow.
- There are no pressure drops in the heat exchangers, condensers, and pipes.
- Heat and friction losses, as well as the change in potential and kinetic energies, are neglected.
- The condenser temperature is 303.15 K, and the saturated liquid is supposed to be at the condenser exit.
- Turbine and pump isentropic efficiencies are 0.8 and 0.7, respectively.
- The pinch temperature difference in the evaporator is 8 °C.
- The environment pressure is 101.35 kPa, and the temperature is 298.15 K

### 2.4 Exergy Analysis and Irreversibility Components

Exergy analysis allows calculating exergy destruction of a system’s components, recognizing the components with high exergy destruction, and finding ways to decrease it. Table 2 presents the exergy destruction equations for different components.

**Table 2:** Exergy Destruction Equations for Different Components

evaporator	*İ*_evap_ = *m*_1_*e*_1_ – *m*_2_*e*_2_ + *m*_in exhaust_ – *m*_out exhaust_*e*_out exhaust_
condenser	*İ*_cond_ = *m*_1_*e*_1_ + *m*_3_*e*_3_ – (*m*_2_*e*_2_ + *m*_4_*e*_4_)
pump	*İ*_pump_ = *m*_1_*e*_1_ – *m*_2_*e*_2_ + *Ẇ*_pump_
turbine	*İ*_turbine_ = *m*_1_*e*_1_ – *m*_2_*e*_2_ – *Ẇ*_turbine_
feed-water heater	*İ*_fw_ = *m*_1_*e*_1_ + *m*_2_*e*_2_ – (*m*_3_*e*_3_)
internal heat exchanger	*İ*_IHX_ = *m*_1_*e*_1_ + *m*_3_*e*_3_ – (*m*_2_*e*_2_ + *m*_4_*e*_4_)
separator	*İ*_separator_ = *m*_1_*e*_1_ + *m*_2_*e*_2_ – (*m*_3_*e*_3_)
throttle valve	*İ*_valve_ = *m*_1_*e*_1_ – *m*_2_*e*_2_
mixer	*İ*_mixer_ = *m*_1_*e*_1_ + *m*_2_*e*_2_ – (*m*_3_*e*_3_)

Based on the second law of thermodynamics, the exergy efficiency of systems can be calculated by eq 1 as follows:
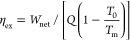
1where *T*_0_ is the ambient temperature, and *T*_m_ is the average temperature of the heat source, which can be calculated as eq 2:
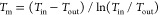
2

### 2.5 Economic Modeling and Thermo-Economic Analysis

#### 2.5.1 Purchase Cost of the Component

For thermo-economic investigations of the relevant cycles, we need a series of relationships to calculate the cost of purchasing the components used in the system. These costs are determined based on system operating conditions and thermodynamic parameters.

The costs of an internal heat exchanger and evaporator are strongly dependent on the heat transfer area. In this regard, to determine the heat transfer area, the overall heat transfer coefficient between hot and cold fluids must be calculated. In this research, we select the overall heat transfer coefficient according to operating conditions and working fluid of the system by reference. Considering the superheater, evaporator, economizer, and heater as heat exchangers, the capital cost of the system components can be calculated by the equations given in Table 3.

**Table 3:** Capital Cost of the System’s Components

evaporator	*Ż*_Evaporator_^CI^ = 130()^0.78^
	*Q*_e_ = *U*_coefficient_*A*_evaporator_Δ*T*_LMTD_
pump	*Ż*_Pump_^CI^ = 3450 (*Ẇ*_Pump_)^0.71^
condenser	*Ż*_Condensor_^CI^ = 1773*m*_f_
turbine	Log_10_(*Ż*_Turbine_^CI^) = 2.629 + 1.4398 log_10_(*Ẇ*_Turbine_) – 0.1776(log_10_(*Ẇ*_Turbine_)^2^)
feed water	*Ż*_Feed – water_^CI^ = *C*_fw_()^1.7^
	log_10_(*C*_fw_) = 4.20–0.204 log_10_(*V̇*_fw_) + 0.1245(log_10_(*V̇*_fw_))^2^

#### 2.5.2 Organic Rankine Cycle Cost Balance

Table 4 presents the exergetic equations with auxiliary equations for three ORCs.

**Table 4:** Exergetic Cost Rate Balances and Corresponding Auxiliary Equations for BORC, SRORC, and DRORC

device	BORC	SRORC	DRORC
evaporator	*Ċ*_4_ + *Ċ*_q eva_ + *Ż*_Evaporator_ = *Ċ*_1_	*Ċ*_4c1a_ + *Ċ*_q eva_ + *Ż*_Evaporator_ = *Ċ*_1_	*Ċ*_4c1a_ + *Ċ*_q eva_ + *Ż*_Evaporator_ = *Ċ*_1_
	*ċ*_q=0.001_	*ċ*_q=0.001_	*ċ*_q=0.001_
turbine	*Ċ*_1_ + *Ż*_Turbine_ = *Ċ*_w tur_ + *Ċ*_2_	*Ċ*_1_ + *Ż*_Turbine_ = *Ċ*_w tur_ + *Ċ*_2a_ + *Ċ*_2c1a_	*Ċ*_1_ + *Ż*_Turbine_ = *Ċ*_w tur_ + *Ċ*_2a_ + *Ċ*_2c1a_ + *Ċ*_2c2a_
	*ċ*_1_ = *ċ*_2_	*ċ*_1_ = *ċ*_2a_, *ċ*_1_ = *ċ*_2c1a_	*ċ*_1_ = *ċ*_2a_, *ċ*_2a_ = *ċ*_2c1a_, *ċ*_2c1a_ = *ċ*_2c2a_
pump	*Ċ*_3_ + *Ċ*_w pump_ + *Ż*_pump_ = *Ċ*_4_	*Ċ*_3c1_ + *Ċ*_w pump1_ + *Ż*_pump1_ = *Ċ*_4c1a_	*Ċ*_3c1_ + *Ċ*_w pump1_ + *Ż*_pump1_ = *Ċ*_4c1a_
	*ċ*_3_ = *ċ*_4_	*Ċ*_3_ + *Ċ*_w pump2_ + *Ż*_pump2_ = *Ċ*_4a_	Ċ_3c2_ + Ċ_w pump2_ + Ż_pump2 =_ Ċ_4c2a_
		*ċ*_3_ = *ċ*_4a_, *ċ*_3c1_ = *ċ*_4c1a_, *ċ*_w pump1_ = *ċ*_w pump2_ = *ċ*_w tur_	*Ċ*_3_ + *Ċ*_w pump3_ + *Ż*_pump3_ = *Ċ*_4a_
			*ċ*_3_ = *ċ*_4a_, *ċ*_3c1_ = *ċ*_4c1a_, *ċ*_3c2_ = *ċ*_4c2a_, *ċ*_w pump1_ = *ċ*_w pump2_ = *ċ*_w pump2_ = *ċ*_w tur_
condenser	*Ċ*_2_ + *Ż*_condenser_ = *Ċ*_q con_ + *Ċ*_3_	*Ċ*_2a_ + *Ż*_condenser_ = *Ċ*_q con_ + *Ċ*_3_	*Ċ*_2a_ + *Ż*_condenser_ = *Ċ*_q con_ + *Ċ*_3_
	*ċ*_2_ = *ċ*_3_	*ċ*_2a_ = *ċ*_3_, *ċ*_q eva_ = *ċ*_q con_	*ċ*_2a_ = *ċ*_3_, *ċ*_q eva_ = *ċ*_q con_
feed water		*Ċ*_4_ + *Ċ*_2c1a_ + *Ż*_feed-water_ = *Ċ*_3c1_	*Ċ*_4c2a_ + *Ċ*_2c1a_ + *Ż*_feed-water1_ = *Ċ*_3c1_
			*Ċ*_4a_ + *Ċ*_2c2a_ + *Ż*_feed-water2_ = *Ċ*_3c2_

#### 2.5.3 Kalina Cycle Cost Balance

As observed in the last section, the SPECO equations were developed for ORCs. Similarly, the equations for three Kalina cycles are shown in Table 5.

**Table 5:** Exergetic Cost Rate Balances and Corresponding Auxiliary Equations for Kalina Cycles

	basic	KCS34	DRORC
evaporator	*Ċ*_7_ + *Ċ*_q eva_ + *Ż*_Evaporator_ = *Ċ*_8_	*Ċ*_11_ + *Ċ*_q eva_ + *Ż*_Evaporator_ = *Ċ*_9_	*Ċ*_12_ + *Ċ*_q eva_ + *Ż*_Evaporator_ = *Ċ*_15_
	*ċ*_q=0.001_	*ċ*_q=0.001_	*ċ*_q=0.001_
separator	*Ċ*_8_ + *Ż*_separator_ = *Ċ*_1_ + *Ċ*_3_	*Ċ*_9_ + *Ż*_separator_ = *Ċ*_1_ + *Ċ*_3_	*Ċ*_5_ + *Ż*_separator_ = *Ċ*_17_ + *Ċ*_20_
turbine	*Ċ*_1_ + *Ż*_Turbine_ = *Ċ*_w tur_ + *Ċ*_2_	*Ċ*_1_ + *Ż*_Turbine_ = *Ċ*_w tur_ + *Ċ*_2_	*Ċ*_15_ + *Ż*_Turbine_ = *Ċ*_w tur_ + *Ċ*_16_
	*ċ*_1_ = *ċ*_2_	*ċ*_1_ = *ċ*_2_	*ċ*_15_ = *ċ*_16_
mixer	*Ċ*_4_ + *Ċ*_2_ + *Ż*_mixer_ = *Ċ*_5_	*Ċ*_4_ + *Ċ*_2_ + *Ż*_mixer_ = *Ċ*_12_	*Ċ*_6_ + *Ċ*_7_ + *Ż*_mixer 1_ = *Ċ*_8_
			*Ċ*_18_ + *Ċ*_21_ + *Ż*_mixer 2_ = *Ċ*_22_
valve	*Ċ*_3_ + *Ż*_valve_ = *Ċ*_4_	*Ċ*_13_ + *Ż*_valve_ = *Ċ*_4_	*Ċ*_20_ + *Ż*_valve_ = *Ċ*_21_
	*ċ*_3_ = *ċ*_4_	*ċ*_13_ = *ċ*_4_	*ċ*_20_ = *ċ*_21_
pump	*Ċ*_6_ + *Ċ*_w pump_ + *Ż*_pump_ = *Ċ*_7_	*Ċ*_6_ + *Ċ*_w pump_ + *Ż*_pump_ = *Ċ*_7_	*Ċ*_1_ + *Ċ*_w pump 1_ + *Ż*_pump 1_ = *Ċ*_2_
	*ċ*_6_ = *ċ*_7_	*ċ*_6_ = *ċ*_7_	*ċ*_1_ = *ċ*_2_
			*Ċ*_9_ + *Ċ*_w pump 2_ + *Ż*_pump 2_ = *Ċ*_10_
			*ċ*_9_ = *ċ*_10_
condenser	*Ċ*_5_ + *Ż*_condenser_ = *Ċ*_q con_ + *Ċ*_4_	*Ċ*_5_ + *Ż*_condenser_ = *Ċ*_q con_ + *Ċ*_6_	*Ċ*_22_ + *Ż*_condenser_ = *Ċ*_q con_ + *Ċ*_1_
	*ċ*_5_ = *ċ*_4_	*ċ*_5_ = *ċ*_6_	*ċ*_22_ = *ċ*_1_
preheater-1		*Ċ*_7_ + *Ċ*_12_ + *Ż*_HE-2_ = *Ċ*_5_ + *Ċ*_10_	*Ċ*_2_ + *Ċ*_16_ + *Ż*_PHE-1_ = *Ċ*_3_ + *Ċ*_17_
		*ċ*_7_ = *ċ*_12_, *ċ*_5_ = *ċ*_10_	*ċ*_2_ = *ċ*_16_, *ċ*_3_ = *ċ*_17_
preheater-2		*Ċ*_3_ + *Ċ*_10_ + *Ż*_HE-3_ = *Ċ*_11_ + *Ċ*_13_	*Ċ*_3_ + *Ċ*_19_ + *Ż*_PHE-2_ = *Ċ*_4_ + *Ċ*_20_
		*ċ*_3_ = *ċ*_10_, *ċ*_11_ = *ċ*_13_	*ċ*_3_ = *ċ*_19_, *ċ*_3_ = *ċ*_20_
interprecooler			*Ċ*_8_ + *Ċ*_10_ + *Ż*_IPC_ = *Ċ*_q IPC_ + *Ċ*_9_ + *Ċ*_11_
			*ċ*_8_ = *ċ*_10_, *ċ*_9_ = *ċ*_11_
recuperator			*Ċ*_11_ + *Ċ*_17_ + *Ż*_Recuperator_ = *Ċ*_12_ + *Ċ*_18_
			*ċ*_11_ = *ċ*_17_, *ċ*_12_ = *ċ*_18_

### 2.6 Optimization

In this work, the “deterministic sampling” method was adopted as the selection operator. The crossover operator is responsible for producing new chromosomes. The simple arithmetic crossover was employed in this research. The mutation operator is used to modify the values of chromosomes randomly to avoid converging to local solutions. The “elite-preservation strategy” was employed to protect the elites from crossover and mutation, thereby accelerating convergence. Configurations of the GA in this work are shown in Table 6.

**Table 6:** Configurations of Genetic Algorithms

individuals	32
objective function	*c*_w_ and ...
chromosome vector	[T_1_,P_1_,X_j_]
mutation probability	0.4
generations	256
iterations	8480

In this work, we use this algorithm in two cases. First, we use it to maximize exergy efficiency by inlet pressure and temperature of the turbine. The mass fraction of flow rate in the turbine is also used in SRORC and DRORC. In the Kalina cycle, the concentration of the ammonia–water mixture is also selected as the parameter to optimize second law efficiency. Second, we aimed to minimize the specific exergy cost of cycles in thermo-economic analysis to find the best condition to work. In this optimization process, the specific exergy cost of power production (*c*_p_) was selected as the fitness function. By minimizing *c*_p_, we can maximize the exergy efficiency of cycles with minimum possible costs. In this research, EES was used because of these features to simulate the cycle process and calculate the efficiency and optimization. MATLAB software was linked with EES, and optimization was performed.

## 3 Results and Discussion

In the above sections, we discussed different organic and Kalina cycles used for waste heat recovery to produce work. We selected three different models of each cycle to study and analyze in this research. In this section, we analyze them with different temperature ranges identified in the steel industry. First, we focus on the optimization and sensitivity analysis of the ORCs and Kalina cycles with sensible heats. Then, we discuss the economics of systems and optimize them from the thermo-economic perspective.

### 3.1 Optimization of Organic Rankine Cycles

As discussed before, we selected two temperature ranges from the steel industry to recover waste heat and produce power. These two temperatures are observed in the rolling section and electric arc furnace of the steel-making section. The following sections present the results for ORCs with optimization conditions.

#### 3.1.1 Waste Heat Recovery in the Rolling Section

Based on the GA, three different ORCs are optimized to maximize exergy efficiency. In this work, we selected four various working fluids, i.e., R245fa, R141b, R123, R11, and R141b. Optimization was done for the first temperature range that is wasted in the rolling section. The turbine inlet temperature and pressure in BORC and, in regenerative cycles, turbine inlet temperature and pressure with fractions of flow rate are selected as optimizing parameters. In Appendix A, Table A1 presents the results and some thermodynamic parameters under optimal operating conditions with working fluids for each system.

**Table A1:** Optimum Condition of ORCs with Different Working Fluids in the Rolling Section

	working fluid											
	R11			R123			R141b			R245fa		
thermodynamic parameter	BORC	SRORC	DRORC	BORC	SRORC	DRORC	BORC	SRORC	DRORC	BORC	SRORC	DRORC
*T*_1_ (K)	411	410	412	393.7	409.7	410	409	410	409.9	388.5	397.2	397.5
*P*_1_ (kPa)	1447	1620	1698	1213	1650	1660	1210	1370	1398	1757	2088	2100
*P*_3_ (kPa)	125.3	125.3	125.3	109.7	109.7	109.7	94.16	94.16	94.16	177.2	177.2	177.2
η_e_	58.76	63.27	65.94	56.55	61.72	64.42	57.71	62.77	65	55.17	58.9	60.17
η_t_	16.14	18.03	18.94	14.52	17.69	18.53	15.76	17.88	18.64	13.28	15.56	16.05
*W*_p1_ (kW)	1.398	0.5853	0.1854	2.562	0.4201	0.293	1.175	0.4628	0.3164	5.246	3.808	3.295
*W*_p2_ (kW)			0.05609			0.1099			0.08635			0.9221
*W*_p3_ (kW)		0.1336	0.02235		0.1031	0.04479		0.1179	0.03532		0.724	0.412
*W*_t_ (kW)	42.4	16.56	5.612	78.2	10.81	8.686	44.76	16.41	11.85	104	68.56	70.17
*W*_net_ (kW)	41	15.85	5.348	75.64	10.29	8.239	43.58	15.83	11.41	98.74	64.03	65.52
*m* (kg/s)	1.084	0.4828	0.1682	2.359	0.3414	0.2952	0.9029	0.3868	0.2829	3.08	2.145	2.329
*Q*_e_ (kJ/s)	254.1	99.93	31.34	520.9	65.45	56.6	276.5	94.61	68.96	743.6	451.9	499
*Q*_c_ (kJ/s)	213.1	87.85	28.24	445.2	58.15	44.46	233	88.52	61.23	644.9	411.5	408.3
*X*_c1_		0.2001	0.1585		0.2687	0.2087		0.2317	0.164		0.2312	0.1844
*X*_c2_			0.1113			0.1514			0.1136			0.1288

**Figure 8:**
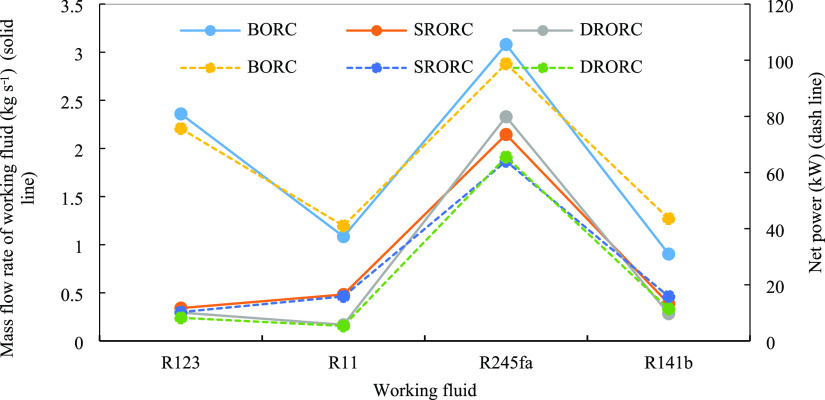
Mass flow rate and net power for cycles in optimal conditions with various fluids.

The results of optimization in the table show that in regenerative cycles, the outlet temperature of the heat source is higher than that of the basic Rankine cycle. It can be explained that in regenerative cycles, we extract some heat from turbine output to preheat the flow of the evaporator, so the amount of heat absorbed from the heat source is reduced and the output temperature is increased. Among the selected working fluids, R245fa recovered more heat from the heat source, so we see a lower output temperature than the other fluids. The amount of heat exchanged in the condenser is another parameter that has been investigated. In regenerative cycles, the amount of heat that is ejected in the condenser is lower than that in the simple ORC because more heat is recovered. Also, the turbine output flow, which is the inlet of the condenser, is lower in regenerative cycles than in the simple cycle, which reduces the demand for cooling compared to the simple cycle. Figure 8 shows the mass flow rate of the cycle for various fluids in optimal conditions. As it is seen in the diagram, the amount of mass required in the regenerative cycles is lower than that required in the organic basic cycle. This is related to the fact that less heat absorption is required in the evaporator of the regenerative cycles. In various cycles, R245fa always requires higher mass flow rates due to its thermodynamic properties.

The net power output for different cycles is shown in Figure 8. In regenerative cycles, the mass fraction that is regenerated reduces the quality of steam used in the turbine, so power generation is decreased in the system. By analyzing this parameter, the R245fa fluid will have the highest production capacity among the selected fluids. The important point in using regenerative cycles is that the increase in the amount of heat that is exchanged exceeds the amount that decreases in output power versus the simple Rankine cycle.

**Figure 9:**
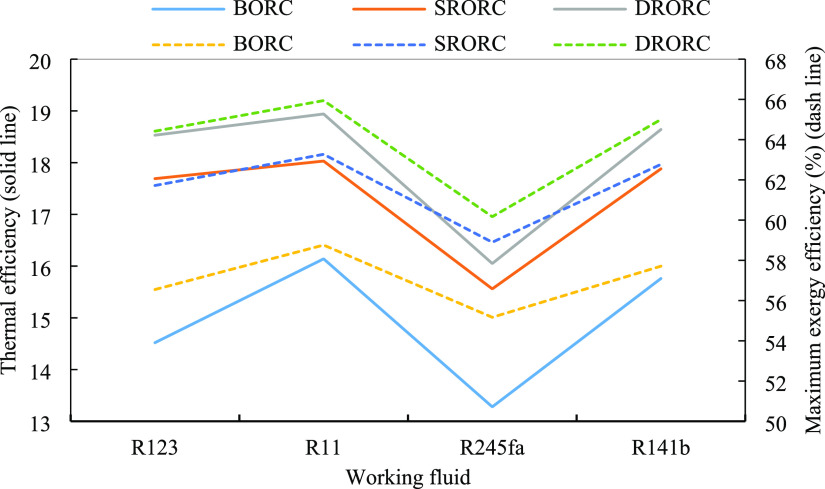
Maximum energy and exergy efficiency for cycles in optimum conditions with various fluids.

Figure 9 displays the thermal efficiency and exergy efficiency for the cycles in optimal conditions with different fluids. The variation of these two efficiencies with fluid type is almost the same. For all fluids, the cycles that have two heat regenerative exchangers are more efficient than the other cycles from the energy and exergy viewpoint.

The R11 and R141b fluids are related to higher efficiency than the other fluids. With an increase in the output temperature of the hot source, the heat that is exchanged in the evaporator reduces, so we observe higher thermal efficiency. In regenerative cycles, reducing the temperature difference between the temperature of the heat source and the working flow inside the system causes a reduction in thermodynamic irreversibility, so the exergy efficiency is increased. According to Figure 9, we observe that among all fluids, R245fa has the lowest efficiency but the highest net output power. Therefore, it should be noted that the power produced is not by itself a suitable parameter for optimizing the systems. It is therefore concluded that regenerative cycles with lower thermal loads have higher efficiency than simple ORCs.

#### 3.1.2 Waste Heat Recovery from EAF

This section deals with optimization for the second temperature range wasted from EAF. The turbine inlet temperature and pressure are selected as optimizing parameters in BORC, and the turbine inlet temperature and pressure with fractions of flow rate are selected as optimizing parameters in regenerative cycles. The results and some thermodynamic parameters under optimal operating conditions with working fluids for each system are listed in Table A2. According to the results, when the temperature and mass flow rate of the heat source are higher, optimal results can be obtained by making small changes in the temperature and output pressure of the turbine. These changes are more influential on the mass of working fluid in the system and increase this parameter in the cycle significantly. This means that if we want to recover heat from heat sources with higher temperatures, we should design cycles that consider more working fluids to have more potential for heat recovery.

**Table A2:** Optimum Condition of ORCs with Different Working Fluids in EAF

	working fluid											
	R11			R123			R141b			R245fa		
thermodynamic parameter	BORC	SRORC	DRORC	BORC	SRORC	DRORC	BORC	SRORC	DRORC	BORC	SRORC	DRORC
*T*_1_ (K)	413.6	411.4	417.1	407.4	414.1	415.1	415	413.2	414.8	399.6	400	400.3
*P*_1_ (kPa)	1651	1719	1863	1583	1692	1819	1554	1493	1539	1797	2156	2214
*P*_3_ (kPa)	125.3	125.3	415.4	109.7	109.7	109.7	94.16	94.16	94.16	177.2	177.2	177.2
η_e_	60.33	55.04	57.04	74.35	55.75	60.63	65.94	56.94	57.32	89.85	59.7	60.74
η_t_	16.6	18.33	19.43	15.66	18.06	18.66	16.74	18.29	19.09	13.37	15.69	16.33
*W*_p1_ (kW)	59.3	52.28	53.4	69.47	68.5	69.81	55.92	56.94	44.38	89.46	98.44	100.4
*W*_p2_ (kW)		12.86	11.7		12.89	9.707		18.29	8.438		17.27	15.2
*W*_p3_ (kW)			5.703			5.084		47.3	4.196			8.169
*W*_t_ (kW)	1556	1401	1415	1771	1615	1666	1667	8.196	1461	1746	1731	1768
*W*_net_ (kW)	1496	1336	1344	1701	1533	1581	1611	1494	1404	1656	1615	1644
*m* (kg/s)	39.92	40.91	40.22	47.9	47.96	47.98	32.82	1436	32.8	51.22	52.66	53.95
*Q*_e_ (kJ/s)	9014	7588	7498	10,862	9226	9238	9624	32.96	8009	12,391	11,183	11,392
*Q*_c_ (kJ/s)	7581	6278	5917	9161	8493	7474	8013	8036	6351	10,735	9297	9065
*X*_c1_		0.2169	0.1391		0.2456	0.1382		6863	0.1412		0.2237	0.145
*X*_c2_			0.1176			0.1157			0.1163			0.1158

At higher temperatures with more flow, R245fa has higher exergy efficiency than the other working fluids. As we discussed, the mass flow rate plays an important role in cycles in higher ranges. So, due to its thermodynamic properties, R245fa needs higher mass and provides higher heat capacity in the system, resulting in the lowest efficiency in low-temperature ranges and the highest efficiency in the middle- and high-temperature ranges.

The remaining parameters have almost the same conditions as was discussed about them in the previous part.

### 3.2 Optimization of Kalina Cycles

The following subsections deal with optimizing the Kalina cycles with waste heat to find best conditions for the working of systems.

#### 3.2.1 Waste Heat Recovery in the Rolling Section

Based on GA, we optimize three different Kalina cycles to maximize exergy efficiency. The concentration of ammonia in the working fluid has a significant effect on system efficiency. For this study, we set the concentration of ammonia at four different amounts.

Optimization was done for the first temperature range wasted in the rolling section. The turbine inlet temperature and pressure in three Kalina cycles are selected as optimizing parameters. Table A3 presents the results and some thermodynamic parameters under optimal operating conditions with different working fluids for each system.

**Table A3:** Optimum Parameters of the Kalina Cycles with Different Ammonia Concentrations in the Rolling Section

	concentration of ammonia											
	0.8			0.85			0.9			0.95		
thermodynamic parameter	basic	KCS34	complex	basic	KCS34	complex	basic	KCS34	complex	basic	KCS34	complex
*T*_1_ (K)	406	406	406.7	409.3	409.3	408.3	410.8	410.8	408.4	411.2	411.2	409.3
*P*_1_ (kPa)	2365	2365	2739	2610	2610	2847	2002	2002	2952	2013	2013	3052
η_e_	44.94	44.94	67.57	50.03	50.03	67.73	53.96	53.96	68.96	65.49	65.49	70.13
η_t_	16.89	16.89	16.21	17.35	17.35	16.34	17.72	17.72	16.39	20.41	20.41	16.61
*W*_p1_ (kW)	2.78	2.78	118.7	3.128	3.128	124.4	2.567	2.567	137.4	2.91	2.91	144.4
*W*_p2_ (kW)			4.336			4.551			4.539			4.925
*W*_t_ (kW)	193.2	193.2	290.7	215.2	215.2	304	239.7	239.7	316.6	280.5	280.5	328.4
*W*_net_ (kW)	190.5	190.5	167.6	212	212	175.1	237.2	237.2	174.8	277.6	277.6	179.1
*m* (kg/s)	0.8626	0.8626	0.9259	0.8385	0.8385	0.8749	0.8834	0.8834	0.8411	0.9536	0.9536	0.8496
*Q*_e_ (kJ/s)	1127	1127	1034	1156	1156	1052	1339	1339	1066	1360	1360	1078
*Q*_c_ (kJ/s)	917.7	917.7	784.4	922.6	922.6	826.6	1162	1162	869.3	1297	1297	959

According to the results, in the complex Kalina cycle, higher pressure is needed in the turbine inlet versus the other two cycles to reach the best performance. In the complex cycle, there are three pressure ranges, and two pumps are used in the system design. By changing the ammonia concentration, the turbine inlet temperature decreases. As seen in the optimization results, when the turbine inlet temperature is constant, higher NH_3_ mass fraction is needed and higher net output power and thermal efficiency are obtained. When the NH_3_ mass fraction is constant, the net output power and thermal efficiency first increased and then decreased with increasing turbine inlet temperature. This is related to the fact that with increasing turbine inlet temperature, the mass flow of the basic ammonia solution is reduced to avoid the temperature cross in the regenerator. On the other hand, the higher the temperature, the higher the enthalpy drop of the ammonia-rich steam. When the turbine inlet temperature is less than 190 °C, the mass flow of the ammonia-rich steam is larger and the enthalpy drop of the turbine is increased, so the net output power increases.

The quantity of heat that is exchanged in the evaporator and condenser in the complex cycle and KCS34 is less than that in the basic Kalina cycle. In the complex cycle and KCS34, we have some recuperators and internal heat exchangers that help recover more heat in the cycle and this raises a need for low loads for cooling and heating.

Energy consumption of the pumps in the basic cycle and KCS34 is almost the same, but it is observed in the complex cycle that pump 1 consumes more power because of more mass flow and higher pressure that are needed in the system.

#### 3.2.2 Waste Heat Recovery from EAF

In this section, optimization is done for the second temperature range wasted from EAF. The turbine inlet temperature and pressure in the Kalina cycles are selected as optimizing parameters. The results and some thermodynamic parameters under optimal operating conditions with different concentrations of ammonia in the working fluid for each system are listed in Table A4. By changing the conditions of the heat source and using higher temperatures with more mass flow, we need higher pressure and temperature in the turbine inlet to reach optimum conditions and have the best performance. The results in this section show that in the Kalina cycles, exergy efficiency reduces with an increase in the temperature of the heat source. Because of the thermodynamic properties of the ammonia–water mixture, we cannot recover more loads from some ranges above and more exergy is destructed, causing lower exergy efficiency.

**Table A4:** Optimum Parameters of Kalina Cycles with Different Ammonia Concentrations in EAF

	concentration of ammonia											
	0.8			0.85			0.9			0.95		
thermodynamic parameter	basic	KCS34	complex	basic	KCS34	complex	basic	KCS34	complex	basic	KCS34	complex
*T*_1_ (K)	469.8	465.5	458.7	471.5	466.3	462.9	475.2	468.6	465.7	478.2	473	474.3
*P*_1_ (kPa)	2823	3060	3218	3354	3564	3426	3956	3973	3571	4697	3957	3492
η_e_	35.09	29.06	57.07	41	34.07	58.47	49.25	40.58	59.31	58.73	49.15	58.4
η_t_	17.71	18.3	19.42	20.86	21.29	19.88	24.66	24.59	20.14	29.11	27.76	19.65
*W*_p1_ (kW)	11.93	11.04	595.6	15.61	14.02	694.3	20.73	17.35	806.5	27.74	19.74	923.6
*W*_p2_ (kW)			26.17			31.18			34.69			36.69
*W*_t_ (kW)	1000	828.6	2080	1170	972.4	2221	1408	1159	2360	1682	1402	2472
*W*_net_ (kW)	988	817.5	1459	1155	958.4	1495	1387	1142	1519	1654	1382	1512
*m* (kg/s)	3.056	2.922	4.644	3.197	3.075	4.884	3.423	3.273	5.169	3.665	3.637	5.533
*Q*_e_ (kJ/s)	7872	4468	7511	7177	4502	7519	7233	4642	7542	7132	4980	7695
*Q*_c_ (kJ/s)	5577	3318	3784	5535	3288	4404	5623	3493	5136	5681	4208	5968

In new conditions, we need more mass flow of working fluid in the system and, with higher heat source temperature, the system shows higher thermal efficiency by producing more power in the turbine. Heat loads in the condenser and evaporator increase significantly, which should be noticed in designing. The remaining parameters have almost the same conditions as was discussed in the previous section.

### 3.3 Sensitivity Analysis of ORCs

This section analyzes the effect of some important parameters on the performance of the system. As observed in the previous section, R11 has higher exergy efficiency among the selected fluids. So, we selected R11 as working fluid to do our analysis. The optimal conditions were considered the selected parameters.

**Figure 10:**
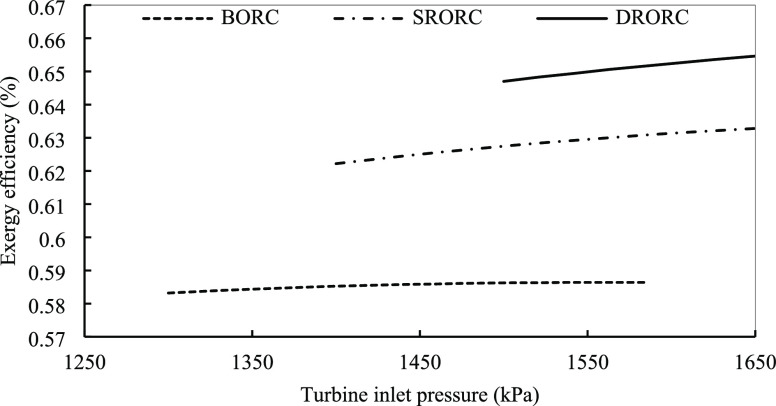
Energy efficiency for ORCs in the optimum condition with turbine inlet pressure.

Figure 10 depicts the effect of turbine inlet pressure on the exergy efficiency of BORC. By increasing the turbine inlet pressure, the pressure ratio of the turbine increases, so the surface under the T-S diagram is increased, implying the production of more work in the system. Also, by increasing the pressure, the total exergy destruction of the system decreases, resulting in higher exergy efficiency in the system. In BORC, at a turbine inlet pressure of above 1550 kPa, we have the same efficiency and the changes can almost be neglected.

**Figure 11:**
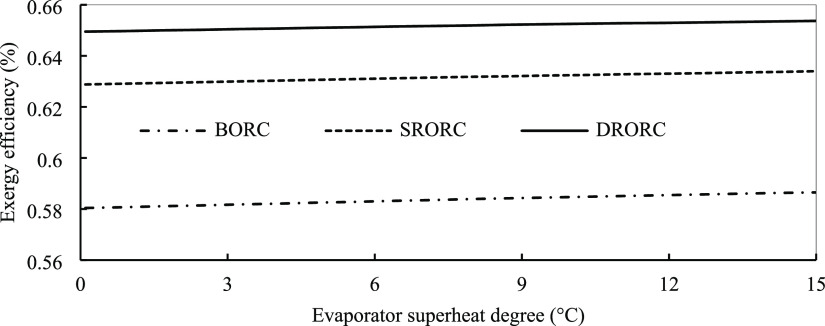
Energy efficiency for BORC, SRORC, and DRORC in optimum conditions with evaporator temperature.

**Figure 12:**
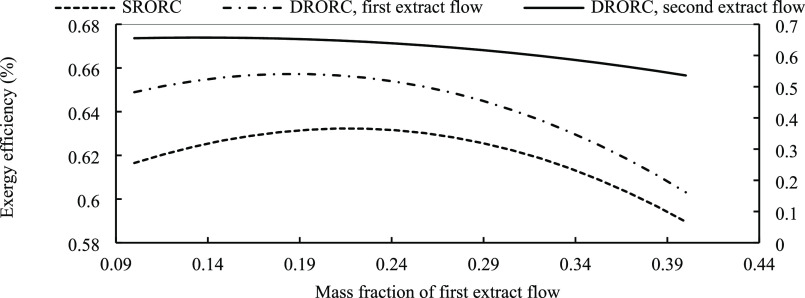
Energy efficiency for SRORC, DRORC, and DRORC in the optimum condition with the extracted flow.

The two above figures show the effect of the turbine inlet pressure on exergy efficiency in SRORC and DRORC. Above the pressures of 1750 and 1850 kPa, SRORC and DRORC almost have the same efficiency. The next parameter selected in this study is the superheat temperature in the turbine inlet. This parameter was analyzed for three ORCs whose results are depicted in Figure 11. It is evident that by increasing the turbine inlet temperature, the exergy efficiency increases slightly. By increasing superheat, more energy goes to the turbine, so we have more changes in enthalpy, which causes more changes in power. Also, from another point of view, increases in the turbine inlet temperature mean that the temperature difference between the evaporator and exhaust gases decreases. So, by this change, we have less exergy destruction in the evaporator, resulting in higher exergy efficiency. As already discussed, in regenerative cycles, we extracted some flow from the turbine outlet to preheat the flow to use the system energy and increase efficiency. The quantity of the mass fraction that is extracted plays an important role in the performance of the cycle. Figure 12 displays the effect of this parameter on cycle efficiency in the single regenerative cycle. The mass quality is an effective variable in optimization. If we extract more mass, the mass flow rate of fluid that enters the turbine blades decreases, so the work that is produced in the system decreases, resulting in lower efficiency. In DRORC, we extracted two flows from the turbine to help recover heat in the cycle. The effect of the first and second mass fractions on the cycle efficiency of DRORC is shown in Figure 12. The quantity of mass extracted first is less than that of the second one, which should be noticed in designing the systems. The optimized mass fraction for SRORC and DRORC is discussed in the last section.

### 3.4 Sensitivity Analysis of Kalina Cycles

**Figure 13:**
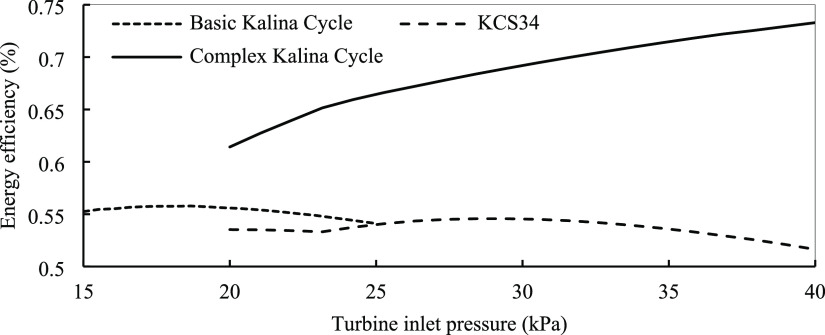
Energy efficiency for the basic, KCS34, and complex Kalina cycles in optimum conditions with turbine inlet pressure.

**Figure 14:**
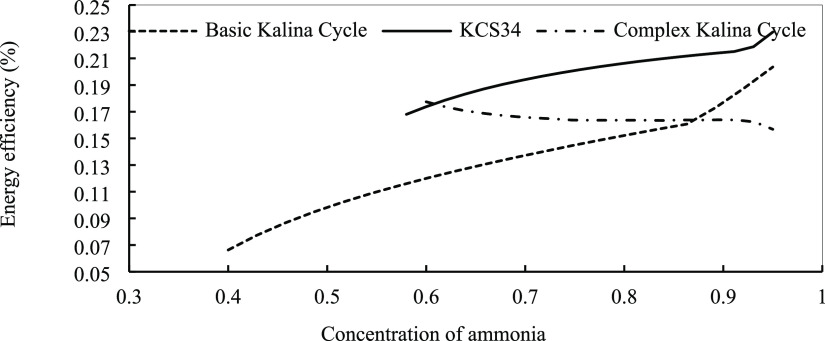
Energy efficiency for the Kalina cycles in optimum conditions with the concentration of ammonia.

**Figure 15:**
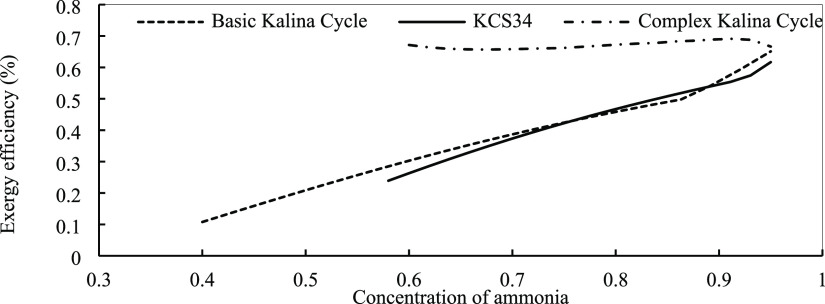
Exergy efficiency for the basic, KCS34, and complex Kalina cycles in optimum conditions with the concentration of ammonia.

**Figure 16:**
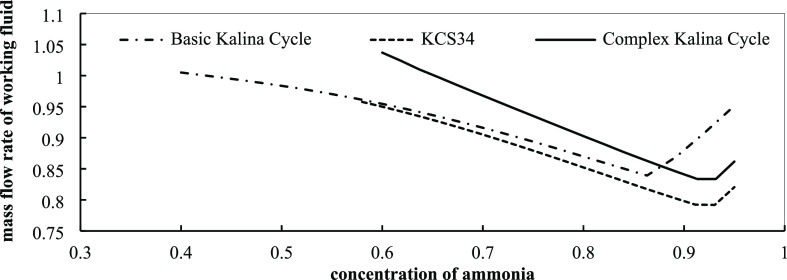
Mass flow rate of the working fluid for the basic, KCS34, and complex Kalina cycles in optimum conditions with the concentration of ammonia.

In this section, we analyze the influence of some important parameters on the performance of the system. Based on the analysis in the previous section, we selected 0.9 as the ammonia concentration for the fluid to do our analysis. To analyze sensitivity to a certain parameter, other parameters are set in optimal conditions to check the changes in that certain parameter. Figure 13 depicts the effect of the turbine inlet pressure on the exergy efficiency of the basic Kalina cycle. As is evident by increasing pressure, the efficiency increases to a level that optimum conditions are reached. By increasing the turbine inlet pressure, the pressure ratio of the turbine increases, so the surface under the T-S diagram increases, which means the production of more work in the system. Also, by increasing pressure, the total exergy destruction of the system decreases, resulting in higher exergy efficiency in the system. But if we further increase pressure in the Kalina cycle, the exergy destruction increases and causes lower efficiency because of the system design and mixture properties. The peak points are formed for two reasons. First, the mass flow rate of vapor indicates the best pressure at different temperatures. The mass flow decreases, so the power generation of the turbine reduces. Second, the power consumption of the working pump increases exponentially, and the net power is reduced at high pressure. For the basic cycle at about 19–20 bars, the system shows the best performance. KCS34 has almost the same performance as the basic one, but in the complex cycle, the efficiency does not decrease at higher turbine inlet pressure. In KCS34 and the complex cycle at 30 and 40 bars, respectively, the system has higher efficiency. Figure 14 illustrates the effect of ammonia concentration in the mixture on the energy efficiency of the basic Kalina cycle and KCS34. As is evident, in both systems, increasing the ammonia concentration results in higher energy efficiency of the systems. It is revealed by the analysis that there is a critical point for the ammonia fraction that should be considered when designing the systems. The effect of mass fraction in the energy efficiency of the complex Kalina cycle is different from the other two cycles. In this cycle, there are two critical points that happen in the fractions of 0.7 and 0.9. In this range, the energy efficiency is almost fixed. Because of the system design, more heat is recovered in the cycle at lower fractions, so higher energy efficiency is obtained, but other parameters should also be considered in their design. We observe that the thermal efficiency is about 16.5% for the complex cycle. Figure 15 shows the effect of the mass fraction of ammonia on energy efficiency. At the next step, we analyze the effect of ammonia concentration on exergy efficiency. In Figure 16, it is observed that exergy efficiency is changed by the mass fraction of ammonia in three Kalina cycles. In the basic Kalina cycle and KCS34, exergy efficiency is increased by increasing ammonia concentration.

In the lower temperature range, the mixture with a higher percentage of ammonia has a great potential for recovering heat and producing power in the system. Since the available heat of the cycle is constant, the efficiency is only related to the net output power. The maximum exergy efficiency values for the basic Kalina cycle and KCS34 in the designing point are 0.5 and 0.6%, respectively. Based on the other analyses and the results, the best fraction for ammonia is 0.9. The exergy change curve by ammonia mass fraction in the complex system is different from the other two cycles. As already explained, two critical points are observed for the complex cycle. By increasing the concentration of ammonia in the mixture, the exergy efficiency increases between the two points. The lowest and highest efficiency values are obtained at the mass fractions of 0.7 and 0.9, respectively. The maximum exergy efficiency is about 0.69%, which happens at the ammonia concentration of 0.9. To conclude, we can say that the complex Kalina cycle shows the best performance among the three cycles at a lower degree in terms of energy and exergy, so it can be used, but we should pay attention to economic aspects, too. The last analysis in this section is related to the effect of the ammonia mass fraction in the mixture on the mass flow rate of the working fluid that we need in the system. As seen in Figure 16, the effect is almost the same in the three cycles.

By increasing the mass concentration of ammonia, the mass flow that is needed in the cycle decreases and the concentration of ammonia reaches about 0.9. This can be attributed to the configuration of the cycles. In fact, since the liquid extract of the low-pressure separator has a lower ammonia concentration, which will be separated in the high-pressure separator, the low ammonia mass concentration in the base stream leads to a small amount of ammonia in the liquid extract, resulting in less mass flow passing through the turbine due to the small vapor quality at the high-pressure separator inlet. The mass flow rate has positive effects on power production, but if it is further increased, the costs of the system components will increase, which needs attention.

## 4 Thermo-Economic Analysis

As observed in the last section, we discuss the energy and exergy efficiency of cycles and analysis and their results. Presently, the economic aspect of projects is very important and should be noticed in the study. So, here, we analyze the system from an economic view, too. We selected the SPECO method for the present study. The SPECO method allows analyzing the system from both exergy and economic views. With the SPECO factor, we can choose the cycle that has the best performance in that condition. Here, we optimize and analyze Orcs. Then, we study Kalina cycles.

### 4.1 Thermo-Economic Optimization of Organic Rankine Cycles

By using the genetic algorithm, thermo-economic optimization of the three ORCs with five different working fluids, i.e., R123, R113, R11, R245fa, and R141b, is done. Based on the results, some thermodynamic parameters in the optimal conditions for each system working are reported in Table A5. By analyzing these results, the R113 fluid has a better performance in terms of thermo-economic properties in BORC versus the other fluids in its optimal pressure and working temperature. R11 is also selected as a suitable fluid in terms of exergy efficiency.

**Table A5:** Thermo-Economic Optimization of ORCs with Different Working Fluids in the Rolling Section

	working fluid														
	R123			R113			R11			R245fa			R141b		
thermodynamic parameter	BORC	BRORC	DRORC	BORC	BRORC	DRORC	BORC	BRORC	DRORC	BORC	BRORC	DRORC	BORC	BRORC	DRORC
*P*_1_ (kPa)	704.5	817.6	838	394	454.6	490	781.4	850.4	819.4	1119	1244	1270	601.9	720.4	706.7
*T*_1_ (K)	368.4	375	376.1	368.8	374.7	378.1	371.2	375.1	373.1	367.7	376.2	374.7	368	376.3	375.2
*X*_c1_		0.1934	0.1703		0.1587	0.1567		0.1644	0.1065		0.1724	0.1491		0.2169	0.1382
*X*_c2_			0.1232			0.1273			0.0989			0.1066			0.1157
eff *T*	11.86	13.74	14.32	11.83	13.58	14.48	12.57	14	14.1	11.41	12.9	13.52	12.05	13.99	14.31
eff ex	53.37	55.88	56.72	52.85	55.1	56.91	53.99	56.18	56.48	53.05	54.32	55.58	53.22	55.7	56.75
*C*_P_ ($/GJ)	7.465	8.671	9.339	7.327	8.296	9.016	7.57	8.834	9.162	7.685	8.766	9.246	7.435	8.788	9.007

**Figure 17:**
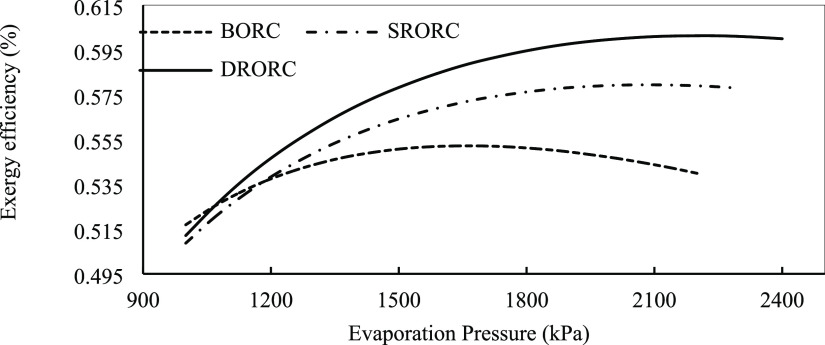
Exergy efficiency of ORCs in optimum conditions with evaporation pressure.

For regenerative ORCs, R113 is also considered the best fluid from the perspective of both thermo-economic and exergy efficiencies. To study the thermodynamic and economic parameters more precisely and to observe the effect of some parameters on cycle performance, R245fa is selected among the five analyzed fluids. For this goal, the R245fa fluid is set in the optimal condition, and the effect of the parameters is shown. Figure 17 displays the effect of turbine input pressure on the exergy efficiency for three systems.

Exergy efficiency increases with increasing evaporation pressure due to the increased production power, and it reaches its maximum value in one point of pressure range. According to Xi et al.,^3^ the pressure is in this range, and a further increase in pressure reduces efficiency.

According to the results, DRORC has the highest efficiency versus the two other systems. SRORC and BORC are in the next ranks. The percentage of mass that is extracted in the two regenerative cycles on turbine output is used to preheat the input flow of the evaporator. This process uses the internal energy of the cycle, which increases the total exergy efficiency of the entire system.

**Figure 18:**
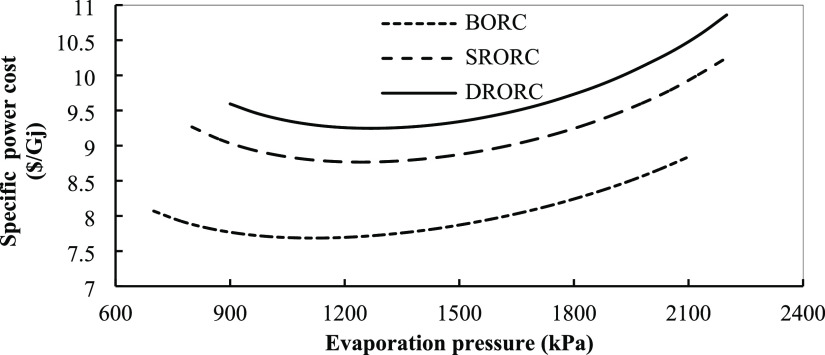
Specific power cost for ORCs in optimum conditions with evaporation pressure.

The optimum pressure to achieve the highest exergy efficiency in BORC is about 1700 kPa, while it is 2100 kPa for SRORC and 2250 kPa for DRORC. The effect of turbine input pressure on the specific power production cost in optimum performance conditions for related systems is shown in Figure 18.

The specific power cost is reduced by increasing the turbine inlet pressure due to an increase in power production. There is a pressure range for each working fluid at which the specific power cost reaches its lowest value. With further increase in turbine input pressure, the costs for heat exchangers will increase and the power generation of the system will decrease. Among the desired cycles, BORC has the lowest specific power cost, and single and double regenerative cycles are in the next ranks.

Also, according to Figure 18, it can be concluded that by accounting for economic issues in the analyses, the optimum turbine inlet pressure is reduced versus the conditions in which only the exergy efficiency is considered in the analysis. By reducing the working pressure, the installation and operation costs of the components are reduced and the systems are optimized from both exergy efficiency and economic aspects.

The effect of superheat temperature in the turbine inlet on the exergy efficiency of the cycles is shown in Figure 17, according to which increasing the superheat temperature will reduce the exergy efficiency.

Increasing the superheat degree at the turbine inlet reduces the mass flow rate of ORCs. By reducing the mass flow rate, the amount of heat that is received in the evaporator will be reduced and this will reduce the output power of the cycle.

**Figure 19:**
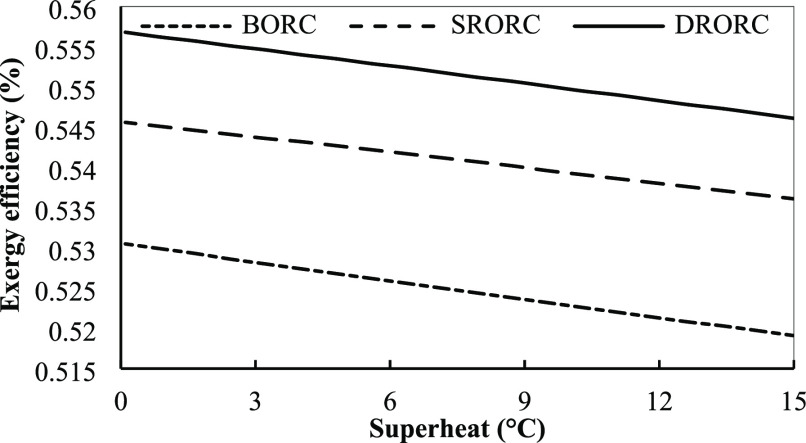
Exergy efficiency for ORCs in optimum conditions with superheat temperature.

**Figure 20:**
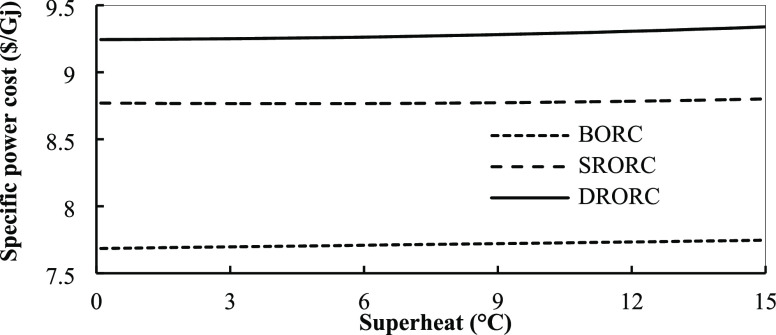
Specific power cost for ORCs in optimum conditions with superheat temperature.

Also, with increasing turbine input temperature, the temperature difference between the heat source and the evaporator will increase, which will increase the irreversibility of the systems and reduce the exergy efficiency. DRORC has the highest exergy efficiency among all cycles as can be seen in Figure 19. The system design type of the regenerative system makes the temperature difference lower between the flow of working fluid and the heat source versus the basic design, and this enhances the efficiency of exergy in these cycle types. Figure 20 shows the effect of superheat temperature in the turbine inlet on the specific power cost. By increasing the superheat degree, the specific power cost increases with a slight slope. To justify this issue, it can be said that by increasing the superheat temperature, the amount of area that is required in the heat exchanger for heat recovery is increased and, since the cost of heat exchangers is a function of their area, these changes will increase their costs. Increasing the cost of heat exchangers will also increase the power production of the systems. Therefore, for BORC, increasing the turbine inlet temperatures will increase system costs like what happens in the regenerative cycles.

Thermo-economic optimization of the three ORCs with five different working fluids is performed by using waste heat of EAF in the steel industry with a genetic algorithm. Based on the results, some thermodynamic parameters in the optimal conditions for each working system are reported in Table A6.

**Table A6:** Thermo-Economic Optimum Solution of ORCs with Different Working Fluids in EAF

	working fluid														
	R123			R113			R11			R245fa			R141b		
thermodynamic parameter	BORC	BRORC	DRORC	BORC	BRORC	DRORC	BORC	BRORC	DRORC	BORC	BRORC	DRORC	BORC	BRORC	DRORC
*P*_1_ (kPa)	2816	3290	2920	2031	2286	2412	3143	2580	2673	2589	2860	2664	2481	2382	2410
*T*_1_ (K)	500.8	500.5	488.9	478.1	470.5	475.7	475.3	501.2	496.6	513.9	497.3	493.1	471.5	485.8	483.9
*X*_c1_		0.2264	0.1824		0.1757	0.1643		0.1875	0.1297		0.1948	0.1715		0.2471	0.1586
*X*_c2_			0.1358			0.1308			0.1102			0.1294			0.1279
eff *T*	17.98	20.52	20.94	18.35	20.66	22.06	19.75	21.07	21.9	14.43	16.3	16.54	18.93	20.83	21.64
eff ex	61.01	59.52	58.76	62.67	62.38	62.47	65.74	58.85	60.2	50.28	49.34	47.37	63.79	57.82	59.42
*C*_P_ ($/GJ)	3.39	3.79	3.98	3.28	3.56	3.74	3.44	3.83	3.93	3.73	4.12	4.42	3.38	3.79	3.82

As results show, the quantity of special power cost decreases by changing the heat source temperature and its mass flow. At higher temperatures, ORC reaches higher efficiency in exergy and energy with more mass flow and this increase compensates for the cost and allows reaching lower special power cost versus lower temperature ranges.

As shown, among the working fluids selected for the study, R113 exhibits the best exergy efficiency and lowest special power cost from thermo-economic analysis in three organic cycles, so it is selected as the best fluid for these conditions. The next ranks are for R113, R141b, R123, and R11 in terms of higher efficiency and lower cost. Because of thermodynamic properties discussed above, R245fa recovers more heat to the cycle and produces more power than the other fluids, but it has lower exergy efficiency.

To reach these conditions, we need exchangers with larger area, but this increases the system cost. In general, R245fa is not suitable for heat recovery and power production in these conditions from a thermo-economic view.

### 4.2 Thermo-Economic Analysis and Optimization of Kalina Cycles

By using the genetic algorithm, thermo-economic optimization of the three Kalina cycles with four different concentrations of ammonia in working fluids is done. The pressure and temperature in the turbine inlet are selected as optimizing parameters, and the goal is to minimize the special power cost of the systems. The thermodynamic parameters in the optimal conditions for cycles and the optimum quantity of pressure and temperature are presented in Table A7.

**Table A7:** Thermo-Economic Optimum Solution of the Kalina Cycles with Different Concentrations of Ammonia in the Rolling Section

	concentration of ammonia											
	0.95			0.9			0.85			0.8		
thermodynamic parameter	basic	KCS34	complex	basic	KCS34	complex	basic	KCS34	complex	basic	KCS34	complex
*T*_1_ (K)	408.5	407.7	403.5	407.3	405.3	401.3	404	402.8	398.4	399	397.5	395.7
*P*_1_ (kPa)	1642	1788	1853	1594	2063	1864	2165	1852	2095	1953	1944	1863
η_t_	18.43	18.23	11.79	15.65	17.98	12.81	15.32	16.39	13.28	13.97	15.4	12.31
η_e_	58.85	65.44	54.96	45.44	57.95	59.09	41.92	52.81	57.19	39.68	48.15	55.42
*C*_P_ ($/GJ)	8.78	6.92	10.37	8.61	6.73	9.93	8.53	6.41	9.84	8.26	6.18	10.09

**Figure 21:**
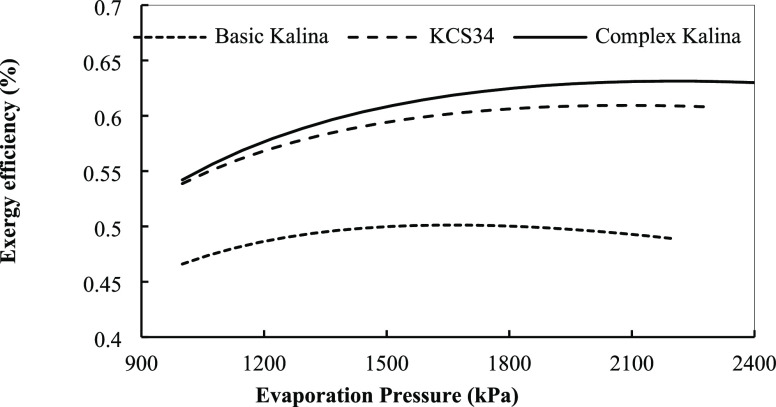
Exergy efficiency for the Kalina cycles in optimum conditions with evaporation pressure.

By analyzing the results of optimization, it is seen that by increasing ammonia concentration in the mixture, the system shows better performance and lower power cost in higher fractions. Since the specific heat capacity of ammonia is smaller than that of water, the vapor flow rate of the ammonia–water mixture generated in the vapor generator increases with increasing ammonia fraction of the ammonia–water mixture. This also leads to an increase in mass flow rate across the ammonia–water turbine, resulting in an increase in turbine power output. The concentration of 0.95 for ammonia was selected as the best fraction for the three cycles from the thermo-economic view. Among the three Kalina cycles, KCS34 has the lowest special power cost in optimized conditions. This means that by this cycle, we can produce more power with lower cost in our conditions that were introduced before. For the basic Kalina cycle, KCS34, and complex cycle, the average special power costs are 8.5, 6.5, and 10 $/GJ, respectively. In KCS34, it is about 24% lower and, in the complex Kalina cycle, it is about 18% higher than that of the basic Kalina cycle. At the next step, the thermodynamic and economic parameters were examined more precisely to observe their effect on cycle performance. For this analysis, the concentration of ammonia is set at the fraction of 0.95, and the conditions are fixed in optimum conditions. Figure 21 presents the effect of turbine input pressure on the exergy efficiency for the three systems.

**Figure 22:**
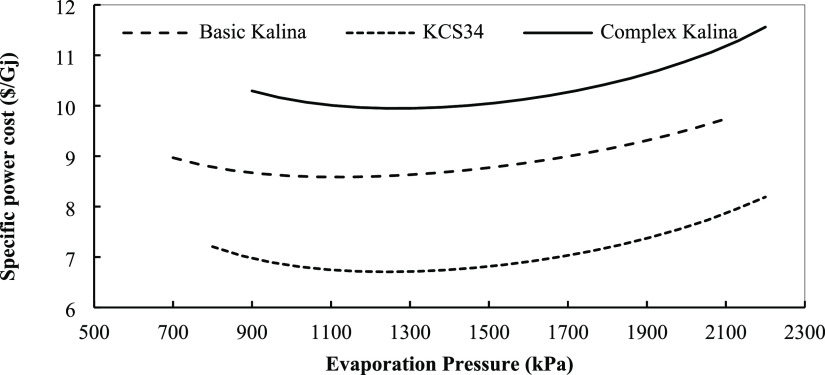
Specific power cost for the Kalina cycles in optimum conditions with evaporation pressure.

As observed in Figure 21, the exergy efficiency of the three Kalina systems increases slightly with increasing evaporation pressure in the system. As the turbine inlet pressure increases, the net power output first increases, reaches its peak, and then starts to decline. It is known that the enthalpy increases in the turbine with increasing turbine inlet pressure, resulting in an increase in turbine power output. By subtracting the pump input from the turbine power output, the net power output increases. This is the reason why the net power output increases at first. But the enthalpy gains from an increasing turbine inlet pressure do not make up for the decrease in mass flow rate of the ammonia–water mixture across the turbine since the vapor flow rate of the ammonia–water mixture generated in the vapor generator decreases with increasing turbine inlet pressure, so the net power output decreases afterward. For the basic cycle, the highest efficiency is obtained at about 1600 kPa, and for the two other cycles, it is obtained at about 2000 kPa in the turbine inlet pressure. The effect of the turbine inlet pressure on the special power cost is analyzed below. Figure 22 displays the variations in the special power cost with evaporation pressure in the three Kalina systems.

**Figure 23:**
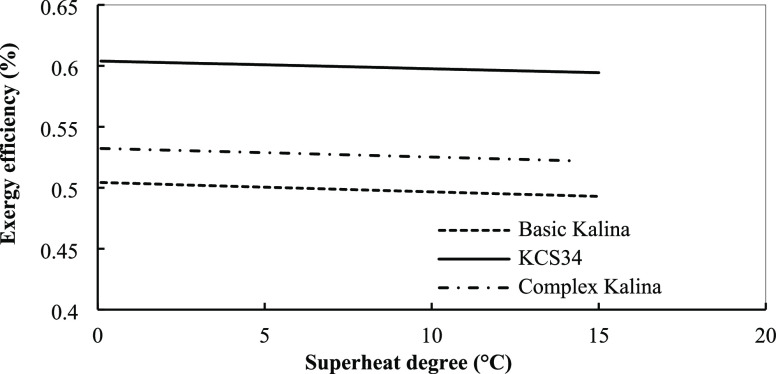
Exergy efficiency for the Kalina cycles in optimum conditions with superheat temperature.

By increasing the turbine pressure, the power cost in the three systems decreases to reach their minimum value. As the turbine inlet pressure increases, the value of heat recovered by the working fluid decreases in the evaporator, but it increases at first and then decreases in the preheater. At low evaporator pressures, the value of heat transfer in the evaporator is higher than that of the preheater, and in general, the total value of heat that gains from the heat source decreases. Also, we can say that at high evaporator pressures, the total value of heat received from the heat source decreases, and with these changes, by increasing the net produced power of the cycle, the thermal efficiency increases, too. By increasing pressure, the investment, operating, and maintenance costs of the components, especially the evaporator and turbine whose costs are dependent on pressure in working conditions, increase. Up to a certain point, if the cost is increased, the increase in exergy efficiency will compensate for the increased cost, but from that point on, the cost will exceed exergy efficiency, resulting in a higher power cost. This point is the optimal point. The optimum pressure range to reach optimum conditions is 1400–2000 kPa for the systems. KCS34 has the lowest value of special power cost among the cycles studied in this research and can be used to recover heat with high efficiency and the lowest possible cost. The effect of superheat temperature in the turbine inlet on the exergy efficiency of the Kalina cycles is shown in Figure 23, according to which by increasing the superheat degree (turbine inlet temperature), the exergy efficiency is reduced.

We can say that by increasing the turbine inlet temperature, the mass flow of the basic ammonia solution will be reduced to avoid the temperature cross in the regenerator. On the other hand, the higher the temperature, the higher the enthalpy drop of the ammonia-rich steam.

**Figure 24:**
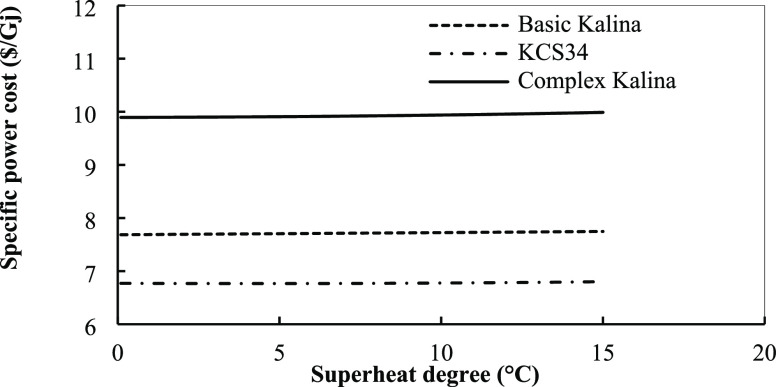
Specific power cost for the Kalina cycles in the optimum conditions with superheat temperature.

At higher temperatures, the effect of mass flow reduction is larger, which decreases the flow of the ammonia-rich steam. Thus, the net output power is reduced. As the total heat of the input system does not change, when the turbine inlet temperature is higher, the heat absorption continues to increase, but the power generated by the turbine decreases, so the thermal efficiency decreases. Also, another reason that was already discussed is that when the superheat degree increases, the mass flow rate of the cycles decreases. By reducing the mass flow rate, the amount of heat that is received in the evaporator will be reduced, and this will reduce the output power of the cycle and exergy efficiency. By this analysis, KCS34 shows the best performance among cycles from this point of view, too. As for the last parameter, the effect of superheat degree on special power cost in the three Kalina cycles is presented in Figure 24. Increasing superheat degree increases the special power cost slightly. When the superheat temperature is increased, components with higher costs will be required to recover heat and produce more power by the turbine for the reasons discussed before. Although increasing superheat degree causes higher efficiency, the costs of components offset this improvement and cause a higher special power cost in the systems. The main effect is exerted by exchangers that need a larger area in these conditions and increase the cost of this component.

## 5 Conclusions

This research mainly aims to recover waste heat in the steel industry for power generation. The steel industry has different sections, which waste some heat in different temperature ranges. In this study, we selected waste heat from the rolling section and electric arc furnace to do our analysis. They are classified into low-temperature and medium-temperature ranges. Three different ORCs, i.e., basic organic Rankine cycle, single regenerative organic Rankine cycle, and double regenerative organic Rankine cycle, are with five different organic fluids selected in this study. Also, for better analysis and the selection of the best cycle, we selected three different Kalina cycles, too. Our analysis is conducted in two main sections: energy and exergy analysis and thermo-economic analysis. At first, we optimize cycles from the view of the first and second thermodynamic laws to find the best cycle for heat recovery in these conditions. In this section, sensitivity analysis of the cycle is conducted, too. In the next step, thermo-economic analysis with the SPECO method has been done to select the system with a lower cost and higher efficiency. The effect of some important parameters on cycle performance is shown, too. In two sections, the inlet pressure and temperature of the turbine and mass fraction in the regenerative cycles are selected as optimizing parameters, and the genetic algorithm is selected as the optimizing method in this research. The important results of this research are listed below: By energy and exergy analysis, the complex Kalina cycle has higher efficiency among all studied cycles with an average of 68% in exergy efficiency. The next ranks are for KCS34 and DROC. In the complex cycle, the optimizing conditions are reached with 3000 kPa and 408 K in the turbine inlet pressure and temperature, respectively. In ORCs, R11 has the highest efficiency among the working fluids and in the Kalina cycle. In general, we can say that the Kalina cycles are suitable to work with a low-temperature heat source, but their efficiency is lower in medium and higher ranges. By using EAF as a heat source, DRORC and SRORC show the best performance and reach high efficiency among selected systems. When using economic analysis, the optimized turbine inlet temperature and pressure are lower than when the thermodynamic analysis is used. The results of optimization show that KCS34 has the lowest special power cost among all cycles with an average of 6.5 $/GJ. The next ranks are for BORC with 7.5 $/GJ and SRORC and basic Kalina with 8.5 $/GJ. By changing the basic Rankine cycle to the single-stage regenerative and double-stage regenerative cycles, 12.5 and 18.75% changes in specific power cost occur with a low heat source, respectively. Working fluids in the cycle play an important role in the performance of systems. R113, R11, and R123 are the best choices for the organic cycle in this study. For the Kalina cycles, the concentration of ammonia is important, and we selected 0.9 to have the highest efficiency based on the results.

Also, the results indicate that in all cycles, as the superheat degree in the turbine inlet increases, the specific power cost increases, and the exergy efficiency of the system decreases. By sensitivity analysis, we found that the highest exergy efficiency and the lowest special power cost happen in some pressure ranges. So, we can say that the turbine inlet pressure and temperature should be noticed when designing systems.

ORCs are the best to recover heat from a medium heat source, and the Kalina cycles show their best performance with low-temperature sources. KCS34, for the rolling section, and DRORC, for EAF heat waste, are selected for heat recovery and more power production in the system.

## Appendix A

Optimum thermodynamic conditions of six cycles in this study are listed in Tables A1–A7.

## Nomenclature

C: condenser
E: evaporator
*e*: specific exergy
*h*: enthalpy
*P*: pressure
*T*: temperature
*Ċ*: stream cost
*e*_ph_: physical exergy
*e*_ch_: chemical exergy
*İ*: irreversibility rate
*ṁ*: mass flow rate
*Q̇*: heat flow rate
*Ẇ*: power
*Ż*: component cost
ε: HE effectiveness
η_e_: exergy efficiency
η_t_: energy efficiency
τ: dimensionless exergetic temperature
BOF: blast oxygen furnace
BORC: basic organic Rankine cycle
DRORC: double regenerative Rankine cycle
EAF: electric arc furnace
FWH: feed-water heater
IHE: internal heat exchanger
in: inlet
KCS: Kalina cycle system
OFC: organic flash cycle
ORC: organic Rankine cycle
out: outlet
PH: preheater
SRORC: single regenerative organic Rankine cycle
SIC: specific investment cost
SH: sensible heat
WHR: waste heat recovery
